# Cod protein powder lowered serum nonesterified fatty acids and increased total bile acid concentrations in healthy, lean, physically active adults: a randomized double-blind study

**DOI:** 10.29219/fnr.v63.3437

**Published:** 2019-03-11

**Authors:** Iselin Vildmyren, Alfred Halstensen, Åge Oterhals, Oddrun A. Gudbrandsen

**Affiliations:** 1 Dietary Protein Research Group, Department of Clinical Medicine, University of Bergen, Bergen, Norway; 2 K. Halstensen AS, Bekkjarvik, Norway; 3 Department of Clinical Science, University of Bergen, Bergen, Norway; 4 Nofima AS, Oasen, Bergen, Norway

**Keywords:** lipid metabolism, protein supplement, residuals, fish protein, cholesterol, lean fish

## Abstract

**Background:**

Fish fillet consumption is associated with beneficial health effects; however, little is known about whether consuming other parts of the fish such as head, backbone, skin, cut-offs, and entrails (collectively known as residuals) will provide comparable effects.

**Objective:**

The aim of the study was to investigate if daily supplementation with cod residual protein powder would impact lipid metabolism in healthy adults.

**Methods:**

Forty healthy, lean, physically active participants (18 women, 22 men) with normal body mass index consumed 8.1 g of proteins daily from cod residual protein powder (Cod-RP) or placebo (control) for 8 weeks.

**Results:**

Cod residual protein powder supplementation lowered fasting serum nonesterified fatty acids and increased serum total bile acid concentrations significantly when compared with control supplementation. Fasting serum low-density lipoprotein cholesterol and apolipoprotein (Apo) B concentrations, as well as the total cholesterol:high-density lipoprotein (HDL) cholesterol and ApoB:ApoA1 ratios, were significantly decreased within the Cod-RP group, but these changes were not different from the control group. Fasting serum concentrations of triacylglycerol, total cholesterol, HDL cholesterol, and ApoA1 were not changed within or between groups.

**Conclusion:**

Eight weeks of daily supplementation with 8.1 g Cod-RP seems to be sufficient to affect lipid metabolism in healthy, lean, physically active adults.

## Popular scientific summary

- Fish fillet consumption is associated with beneficial health effects, however, little is known if consuming the fish residuals (head, backbone, skin, cut-offs and entrails) will provide comparable effects.
- We investigated if daily supplementation with cod residual protein powder would impact lipid metabolism in healthy, lean, physically active adults.
- Cod residual protein supplementation (8.1 g/day) for eight weeks reduced fasting nonesterified fatty acid and increased total bile acid serum concentrations when compared with Control (placebo) supplementation.

## 

The burden of cardiovascular disease (CVD) is one of our largest health challenges globally (1). Certain dietary patterns, such as a high intake of fish, are associated with reduced risk of developing disturbances in lipid metabolism, which in turn reduces the risk of CVD (2–4). High physical activity level is also associated with a lower risk of CVD development through beneficial effects on lipid metabolism (5, 6). Fish and seafood are sources of long-chain n-3 polyunsaturated fatty acids (PUFAs), which have been emphasized when explaining their cardioprotective effects, whereas the possible contributions of other marine nutrients such as proteins have often been overlooked. Recently, several clinical studies have demonstrated that intake of lean fish such as cod, which has a low long-chain n-3 PUFA content but a high protein content, is associated with beneficial effects on circulating metabolites related to lipid metabolism. These effects include higher circulating high-density lipoprotein (HDL) cholesterol (7, 8), lower low-density lipoprotein (LDL) cholesterol (9), and lower triacylglycerol (TAG) concentrations (7, 8, 10, 11). Further, cod fillet intake has showed a tendency to reduce nonesterified fatty acid (NEFA) concentrations in normal-weight adults (12). Cholesterol and NEFA lowering effects have also been demonstrated in obese rats that were fed diets containing proteins from cod fillet (13–16).

Both the cod fillet and residuals from cod fillet production (head, backbone, skin, cut-offs, and entrails) contain high quality proteins (17). Supplementation with cod residual protein powder reduced serum NEFA concentration in overweight and obese adults (18) and protein hydrolysates from residuals of other fish species have shown cholesterol lowering effects in rats (19, 20). However, cod supplement intervention studies in lean and/or physically active adults are, to our knowledge, lacking from the literature. New technologies have allowed cod residuals to be processed efficiently onboard the fishing vessels, resulting in high quality fishmeal powder with high protein content of 60–70% of the dry weight (21). Cod residual fishmeal is currently utilized for fish and animal feeds and should be further exploited for human consumption as a safe and high-quality protein source, which could generate added-value products for the industry.

The aim of the present study was to investigate the impact of daily supplementation with cod residual protein powder (Cod-RP) on lipid metabolism in lean, physically active adults. Our hypothesis was that 8 weeks of daily supplementation with 8.1 g protein from Cod-RP would beneficially affect serum concentrations of lipids and NEFA in lean, physically active adults when compared to a similar group receiving control capsules.

## Materials and methods

### Participants

The current study cohort consisted of lean, physically active adults. Participants were recruited through local newspapers, the intranet at Haukeland University Hospital (Bergen, Norway), the project’s website, social media, and posters in and around Bergen. Recruitment for the study took place between December 2015 and March 2016, and the intervention was conducted between February and June 2016. All participants provided written informed consent before they were enrolled in the study.

The eligibility criteria were as follows: age between 20 and 55 years, fasting blood glucose <7.0 mmol/L, body mass index (BMI) ≥ 18.5 kg/m^2^, and a stable exercise program including two or more training sessions per week. Eligibility criteria also included a stable body weight with less than 5 kg fluctuation in the last 3 months and body fat percentage between 12 and 35% for women and between 5 and 25% for men. Exclusion criteria were as follows: known diseases affecting insulin secretion, heart, intestinal, or kidney function; use of prescribed medications for high cholesterol; allergies towards seafood, milk, or gluten; and tobacco use exceeding 10 cigarettes or snus/day. Candidates taking dietary supplements, consuming >200 g/week of fish and seafood, and pregnant or lactating women were not included in the study.

### Design, intervention, and study protocol

This randomized, double-blind intervention study with a parallel design was conducted at the University of Bergen (Bergen, Norway). Fifty participants were included in the study and stratified according to gender and age by the project manager. The participants consumed capsules containing Cod-RP from Northeast Atlantic cod (*Gadus morhua*) or placebo capsules (control). Participants in the Cod-RP group consumed 27 capsules daily (three doses), providing 8.1 g of cod protein, and participants in the control group consumed 27 capsules per day (three doses) devoid of cod protein. To ensure double-blinding, the capsules were coded by the manufacturer. We assessed compliance by interviewing participants at 4 weeks after study start and at the end point visit. Participants were encouraged to report any deviations from the study protocol.

Before the study, candidates were screened for eligibility by measuring fasting blood glucose concentration with a Contour blood glucose meter (Bayer Consumer Care AG, Basel, Switzerland), height by using a stadiometer (Telescopic Measuring Rod MZ10023-3, ADE, Hamburg, Germany), and body composition by using a bioelectrical impedance analyzer (InBody 720, Seoul, Korea). During the study period, participants were instructed not to make changes in their habitual diet or level of physical activity. The participants attended two study visits, at baseline and after 8 weeks. Both study visits were conducted in the morning between 7 and 11 AM after an overnight fast (>10 h), with no intake of food or drink except water, and no use of medications or tobacco. Participants were also instructed to avoid intensive physical activity and alcohol 24 h before study visits. Body composition was measured at baseline and end point. Fasting blood samples were collected at baseline and end point using Vacutainer SST II Advanced Plus (Becton, Dickinson and Company, Franklin Lakes, NJ, USA) for serum isolation. Serum samples were stored at –80°C until analysis.

### Analysis of estimated dietary intake

Participants recorded their diet for 5 consecutive days, including at least 1 weekend day, prior to the baseline and end point visits. Dietary food records were provided to the participants, and they were instructed to give detailed information about the food and drink they consumed, including descriptions of weight or volume. The participants’ intake of energy and macronutrients were estimated using the dietary assessment software Dietist XP version 3.2 (Kost och Näringsdata, Stockholm AB, Sweden). Protein content from the intervention capsules was included in the estimation of protein intake in the Cod-RP group at the end point registration.

### Production and analysis of intervention capsules

The cod used in the present study were caught and processed onboard the factory trawler Granit (Halstensen Granit AS, Bekkjarvik, Norway). Cod residuals from the cod fillet production were ground, heat treated, and pressed to separate the aqueous fraction (stickwater) from the solid phase (press cake). The solid phase was dried to a press cake meal onboard the trawler, while the stickwater was frozen, transported to land, thawed, and concentrated before it was mixed with the press cake meal and dried to a protein powder (Seagarden AS, Karmøy, Norway).

The capsules were produced by Pharmatech AS (Fredrikstad, Norway). The Cod-RP capsules contained 474.3 mg of Cod-RP, 5.3 mg of magnesium stearate, and 5.3 mg silica per capsule, and the control capsules contained 4.6 mg of each of the two processing aids per capsule. Microcrystalline cellulose was used as stabilizer in the Cod-RP capsules (42.2 mg/capsule) and as replacement for cod residual powder in the control capsules (454.0 mg/capsule). Analyses of amino acids, taurine, and fatty acids in the intervention capsules were conducted by Nofima BioLab (Bergen, Norway).

### Analyses of serum

Concentrations of total bile acids (TBA) and NEFA in serum were analyzed on the Cobas c 111 system (Roche Diagnostics GmbH, Marburg, Germany) using the Diazyme TBA Assay (BioPacific Diagnostic Corporation, Bellevue, WA, USA) and the NEFA FS kit (Diagnostics Systems, Holzheim, Germany), respectively. Total cholesterol, LDL cholesterol, HDL cholesterol, and TAG concentrations in serum were analyzed on the Cobas c 111 system using the appropriate kits from Roche Diagnostics. Apolipoprotein (Apo) A1 and ApoB were analyzed by routine methods at the Laboratory of Clinical Biochemistry at the Haukeland University Hospital (Bergen, Norway).

### Outcome and sample size calculation

The outcome of this study was changes in serum markers of lipid metabolism in healthy, lean adults after 8 weeks of supplementation with Cod-RP. A sample size calculation based on power analysis for the study was not feasible because, to the best of our knowledge, the effects of supplementation with Cod-RP on lipid metabolism have not before been investigated in lean, physically active adults. Two studies with resemblance to the current study have investigated the effects of 8 weeks of protein supplements from cod fillet (9) or cod residuals (18) on glucose regulation, NEFA concentration, and/or lipid metabolism in adults with overweight or obesity. In these studies, between 12 and 18 participants in each group were enough to discover measurable effects. However, the studies used a lower dose (3 or 6 g/day) of cod protein and an obese/overweight population, whereas the current study used a higher dose of 8.1 g/day of cod protein and a lean population with high physical activity. The current study is therefore considered a pilot that contributes to building knowledge in a new field of nutrition research and in generating new hypotheses. The target for the current study was to recruit 50 participants (25 in each group).

### Statistical analyses

The statistic program SPSS Statistics version 25 (IBM Corp. IBM SPSS for Windows, Armonk, NY, USA) was used to perform statistical analysis. The 40 participants who completed the study in accordance with the protocol were included in the statistical analysis. Variables were evaluated for normality by the Shapiro–Wilk test, Q-Q plots, and histograms. Most variables were within normal distribution. Variables that were not normally distributed were log-transformed before parametric statistical tests were performed. Baseline-to-end point changes within groups were tested using the paired-samples *t*-test. The independent samples *t*-test was used to compare changes between the Cod-RP group and the control group. A *p*-value less than 0.05 was considered statistically significant.

## Results

### Participant characteristics

**Fig. 1:**
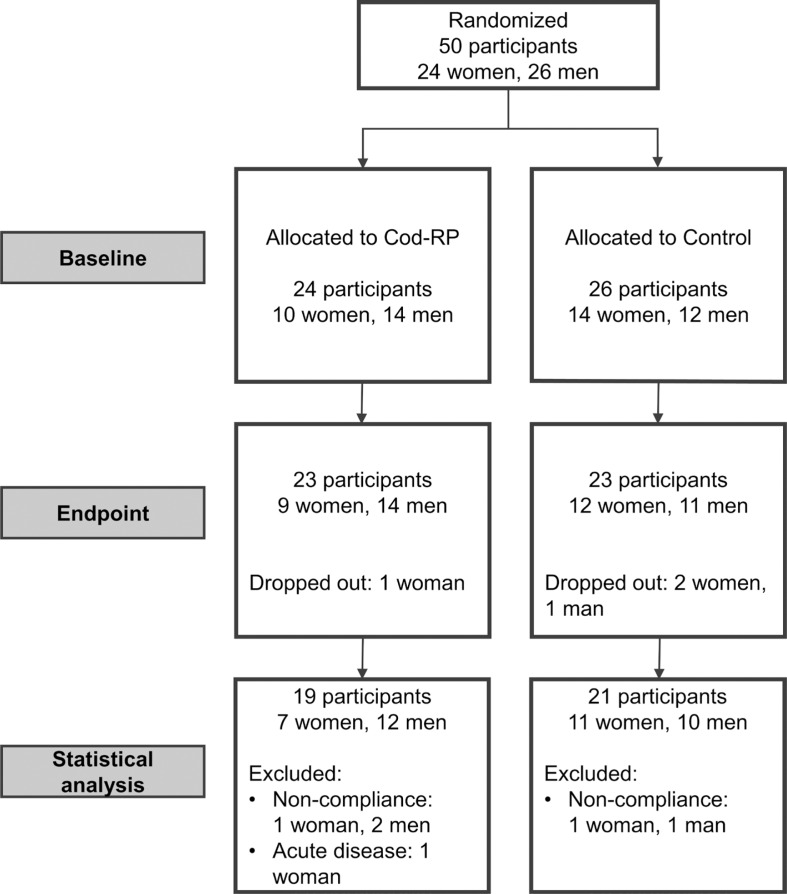
This flow diagram displays the progress of participants through the study. If participants did not comply with the study protocol, they were excluded from statistical analyses. We defined noncompliance as not following the study protocol regarding dietary changes (including fish intake), changes in physical activity habits, and not taking the intervention capsules. Cod-RP, cod residual protein powder.

Fifty participants, 24 women and 26 men, were recruited and randomized to one of the two experimental groups, and of these, 40 participants completed the intervention period according to the study protocol. Four participants (8%) withdrew from the study (one from the Cod-RP group and three from the control group). Six participants (12%) were excluded from statistical analysis due to noncompliance (*n* = 5) or acute disease (*n* = 1). Noncompliance was defined as not following study protocol, which included failing to take the intervention capsules, initiating use of mediations, or implementing dietary changes that could incriminate the study outcome. Participant flow during the study is presented in Fig. 1. Age, BMI, body weight, body fat percentage, body muscle percentage, and tobacco use were similar between groups at baseline (Table 1). BMI, body weight, body fat percentage, and muscle mass percentage were not changed within or between the two intervention groups from baseline to end point (data not presented).

**Table 1:** Participant characteristics at baseline

	Cod residual protein powder (*n* = 19)		Control (*n* = 21)		*p*	
	Mean	Standard deviation	Mean	Standard deviation
Women/men	7/12		11/10		0.36
Age (years)	28.0	6.9	30.5	7.2	0.28
Body weight (kg)	77.0	16.0	73.1	11.5	0.39
Body mass index (kg/m^2^)	24.8	2.8	23.8	2.3	0.25
Fat (%)	19.7	6.8	19.4	6.7	0.89
Muscle (%)	45.4	4.7	45.4	4.4	0.95
Tobacco user (*n*)	1		2		1.00

Note: Results are presented as mean and standard deviation. Groups were compared at baseline using Pearson’s chi-square test for categorical data and independent samples *t*-test for continuous data.

### Concentrations of total bile acids, nonesterified fatty acids, and lipids in serum

The serum NEFA concentration was significantly reduced and the serum TBA concentration was significantly increased in the Cod-RP group compared with the control group after the 8 week intervention (Table 2).

**Table 2:** Total bile acids, lipids, and nonesterified fatty acids in serum

	Baseline		8 weeks		*p*†	*p*‡
	Mean	Standard deviation	Mean	Standard deviation
Total bile acids (mmol/L)						0.0081
Cod residual protein powder (Cod-RP) group	2.68	1.46	3.91	2.21	0.0023	
Control group	2.92	1.92	2.72	1.38	0.83	
Total cholesterol (mmol/L)						0.81
Cod-RP group	4.82	0.85	4.73	0.86	0.24	
Control group	4.68	0.66	4.54	0.61	0.34	
Low density lipoprotein (LDL) (mmol/L)						0.99
Cod-RP group	2.90	0.77	2.74	0.74	0.0061	
Control group	2.66	0.57	2.52	0.61	0.18	
High density lipoprotein (HDL) (mmol/L)						0.16
Cod-RP group	1.68	0.48	1.73	0.54	0.33	
Control group	1.79	0.45	1.74	0.37	0.32	
Total cholesterol:HDL						0.46
Cod-RP group	3.08	0.99	2.94	0.91	0.039	
Control group	2.75	0.76	2.70	0.63	0.75	
ApoA1 (g/L)						0.22
Cod-RP group	1.54	0.27	1.57	0.31	0.25	
Control group	1.61	0.29	1.60	0.26	0.61	
ApoB (g/L)						0.79
Cod-RP group	0.79	0.17	0.76	0.16	0.041	
Control group	0.74	0.13	0.71	0.16	0.47	
ApoB:ApoA1						0.56
Cod-RP group	0.53	0.17	0.50	0.16	0.026	
Control group	0.47	0.15	0.46	0.14	0.44	
Nonesterified fatty acids (mmol/L)						0.0032
Cod-RP group	0.60	0.33	0.52	0.25	0.084	
Control group	0.46	0.20	0.61	0.26	0.012	
Triacylglycerol (mmol/L)						0.24
Cod-RP group	0.81	0.33	0.84	0.31	0.55	
Control group	0.78	0.25	0.74	0.34	0.20	

Note: Results are presented as mean and standard deviation. Serum indicators of lipid regulation are presented for 19 participants in the Cod-RP group and 21 participants in the control group.

† Within-group differences were compared using a paired-samples *t*-test.

‡ Between-group changes were compared using the independent samples *t*-test. The level of significance was set to <0.05.

Fasting serum LDL cholesterol and ApoB concentrations, as well as total cholesterol:HDL cholesterol and ApoB:ApoA1 ratios, were significantly decreased within the Cod-RP group, but these changes were not significantly different from the control group. Serum concentrations of TAG, total cholesterol, HDL cholesterol, and ApoA1 were not significantly changed within or between the groups after 8 weeks of supplementation.

### Estimated macronutrient intake

The participants’ dietary intake was reported prior to the baseline and end point visits. Estimated intake of energy, fat, and carbohydrate were not changed within or between the groups after 8 weeks of intervention (Table 3). The increased estimated protein intake within the Cod-PR group from baseline to end point corresponded with the protein content of these capsules (8.1 g/day), but this change in protein intake was not significantly different when compared to change within the control group.

**Table 3:** Estimated daily energy and macronutrient intake

	Baseline		8 weeks		*p*†	*p*‡
Mean	Standard deviation	Mean	Standard deviation
Energy intake,^1^ kcal/day						0.072
Cod residual protein powder (Cod-RP) group	2,446	681	2,529	665	0.37	
Control group	2,430	486	2,270	469	0.10	
Protein^1^, g/day						0.69
Cod-RP group	122	39	130	38	0.043	
Control group	112	31	118	31	0.28	
Fat, g/day						0.33
Cod-RP group	100	43	96	36	0.56	
Control group	104	30	92	23	0.089	
Carbohydrates, g/day						0.056
Cod-RP group	238	85	261	79	0.084	
Control group	236	62	219	59	0.28	

Note: Results are presented as mean and standard deviation. Estimated daily energy and macronutrient intake are presented for 18 participants in the Cod-RP group and 21 participants in the control group.

† Within-group differences were compared using a paired-samples *t*-test.

‡ Between-group changes were compared using the independent samples *t*-test. The level of significance was set to <0.05.

^a^Energy and protein intake from the capsules (32 kcal/day, 8 g/day protein) were included to the end point – dietary record.

### Daily intake of amino acids, taurine, and fatty acids from capsules

The daily intakes of indispensable amino acids, the nonessential amino acid glycine, the organic acid taurine, and the fatty acids 20:5 n-3 (EPA) and 22:6 n-3 (DHA) from the capsules are presented in Table 4. The daily intake of the long-chain n-3 PUFAs EPA and DHA from the Cod-RP capsules was 87 mg/day. Amino acids, taurine, EPA and DHA were not detected in the control capsules.

**Table 4:** Daily intake of indispensable amino acids, glycine, taurine, and fatty acids from intervention capsules^a^

mg/day	Cod residual protein powder	Control
Isoleucine	341.82	<LOD (level of detection)
Leucine	590.94	<LOD
Lysine	619.40	<LOD
Methionine	256.30	<LOD
Phenylalanine	313.36	<LOD
Threonine	363.10	<LOD
Tryptophan	91.84	<LOD
Valine	384.52	<LOD
Glycine	683.38	<LOD
Taurine	44.14	<LOD
Eicosapentaenoic acid	39.92	<LOD
Docosahexaenoic acid	47.16	<LOD

a Based on the average of two measurements with deviations <5% between parallels.

## Discussion

Here we show that 8 weeks of cod residual protein supplementation (8.1 g/day) lowered serum concentration of NEFA and increased serum TBA concentration compared with placebo supplementation, suggesting that cod residuals may have effects beyond being a source of proteins. We have previously investigated the effects of cod protein supplements on serum markers of lipid metabolism in overweight or obese adults (9, 18). To our knowledge the current study is the first to investigate effects of supplementation with cod residual protein on lipid metabolism in lean adults.

NEFA is involved in lipid metabolism (22); an elevated concentration of NEFA in the circulation is associated with obesity and may also be increased in dyslipidemic individuals (23). In the present study with lean, physically active adults, we observed a significant decrease in serum NEFA concentration in the Cod-RP group when compared to the control group. The current findings in NEFA is in line with a tendency of lower fasting NEFA concentration in normal-weight adults after consuming 750 g/week of cod fillet (12) and lower postprandial NEFA concentration in overweight or obese adults after cod residual protein supplementation (6 g/day) (18).

Reduced serum NEFA concentration and increased gene expressions of the lipogenic enzymes diacylglyceride acyltransferase types 1 and 2 in subcutaneous adipose tissue biopsies have been observed after cod residual protein intake in overweight or obese adults (18), thus suggesting that lower serum NEFA concentration in the current study could be a consequence of inhibition of NEFA release from adipose tissue. In line with this, lower NEFA concentration has also been observed when obese Zucker fa/fa rats were fed cod protein as 25% of their daily protein intake (14). Thus, a lowering effect of cod proteins on serum NEFA concentration seems to be a consistent finding and may be part of the beneficial effects on CVD risk markers associated with lean fish consumption (24, 25).

The increased serum TBA in the Cod-RP group may be a result of increased conjugation of bile acids with glycine and taurine (26, 27). The daily intake of glycine and taurine from the Cod-RP capsules was 683.4 mg and 44.1 mg, respectively, and although this contribution of glycine and taurine was relatively low we cannot exclude the possibility that they may have contributed to increase the concentration of conjugated bile acids, thus leading to increased serum TBA concentration. The main bile acid species associated with improved metabolic health are the primary and secondary glycine-conjugated bile acids (27). However, without quantification of individual bile acid species we cannot determine which of the specific bile acid species contributed to the increased circulating TBA concentration in the Cod-RP group, and quantification of individual bile acid species would be of interest in future studies. The increased serum TBA concentration after Cod-RP supplementation could also be a result of more cholesterol being used for primary bile acid synthesis (26), as serum LDL cholesterol concentration was decreased within the Cod-RP group. This is in line with rat studies where intake of protein hydrolysates of residual materials from saithe or salmon resulted in higher circulating bile acid concentrations (28, 29), but to our knowledge this has not been reported as an effect of fish or fish protein intake in humans. The observed increase in serum TBA concentration after Cod-RP supplementation is of interest because a higher circulating TBA concentration has been associated with improved metabolic health after weight loss following gastric bypass surgery in obese adults (27, 30), and drugs that increase bile acid production can be used to lower LDL cholesterol (31).

Cross-sectional and follow-up studies have observed associations between intake of lean fish and lower serum TAG (8, 11) and increased HDL-cholesterol concentrations (8), none of which were observed in the current study. Randomized controlled trials with cod protein supplements or cod fillet intake have found contrasting results. In overweight or obese adults, cod supplements reduced the LDL cholesterol concentration (9), which was also observed in the current study, albeit only within the Cod-RP group. However, in a recent study no changes in concentrations of TAG, HDL cholesterol, or LDL cholesterol were observed after consumption of 750 g/week of cod fillet in lean adults (12). This may suggest that a high frequency of cod protein intake in the supplement studies with three doses per day, as opposed to a lower frequency with five cod fillet portions per week, may have more potent effects on lipid metabolism. This is also supported by rat studies where lower serum lipid concentrations were observed in obese Zucker rats with free access to cod protein 24 h/day (14, 16). A frequent cod protein intake may have contributed to maintaining a steady state where amino acids or other compounds in the cod protein could lead to a more constant influence on lipid metabolism.

In addition to a high content of protein, the Cod-RP capsules in the current study also contain the long-chain n-3 PUFAs EPA and DHA, which could exert beneficial health effects (32, 33). However, the contribution of EPA and DHA from the Cod-RP capsules was 87 mg/day, which is low compared with the general recommended intake of >250 mg/day of EPA and DHA based on observed protective effects on cardiovascular heart disease mortality, with even higher doses necessary to find effects on circulating markers of lipid metabolism (33). Hence, EPA and DHA intake from the Cod-RP capsules probably had insignificant or no effects on the decrease in serum NEFA and increase in serum TBA concentrations observed in the Cod-RP group.

The current study has some limitations. We did not have the necessary information to perform a sample size calculation in this pilot study because, to the best of our knowledge, no studies have been conducted or published on the effects of cod residual protein intake in a group of healthy, lean, physically active adults. Hence the sample size was determined based on experiences from previous similar clinical studies in overweight or obese adults, which may increase the risk of effects not being discovered (type 2 error), if the sample size was too small.

To conclude, 8 weeks of daily supplementation with 8.1 g Cod-RP seems to be sufficient to affect lipid metabolism in healthy, lean, physically active adults.
